# SMAD4 inhibits glycolysis in ovarian cancer through PI3K/AKT/HK2 signaling pathway by activating ARHGAP10

**DOI:** 10.1002/cnr2.1976

**Published:** 2024-01-17

**Authors:** Kui Wu, Wei Gong, Huanmei Sun, Wenjiao Li, Li Chen, Yingchun Duan, Jianlong Zhu, Hu Zhang, Huihui Ke

**Affiliations:** ^1^ Department of Obstetrics and Gynecology, Shanghai Pudong Hospital Fudan University Pudong Medical Center Shanghai PR China

**Keywords:** ARHGAP10, glycolysis, hexokinase‐II, lactate, ovarian cancer

## Abstract

**Background:**

ARHGAP10 is a tumor‐suppressor gene related to ovarian cancer (OC) progression; however, its specific mechanism is unclear.

**Aims:**

To investigate the effect of ARHGAP10 on OC cell migration, invasion, and glycolysis.

**Methods and Results:**

Quantitative real‐time PCR (qRT‐PCR) quantified mRNA and protein expressions of AKT, p‐AKT, HK2, and SMAD4 were tested by Western blot. EdU, Wound healing, and Transwell assay were utilized to evaluate OC cell proliferation, migration, and invasion. We used a Seahorse XF24 Extracellular Flux Analyzer to monitor cellular oxygen consumption rates (OCR) and extracellular acidification rates (ECAR). Chromatin immunoprecipitation (ChIP) was used to analyze the transcriptional regulation of ARHGAP10 by SMAD4. ARHGAP10 expression in OC tissues was detected by immunohistochemistry. Our results showed that ARHGAP10 expression was negatively related to lactate levels in human OC tissues. ARHGAP10 overexpression can inhibit the migration, proliferation, and invasion of OC cells, and this function can be blocked by 2‐Deoxy‐D‐glucose. Moreover, we found that ARHGAP10 expression can be rescued with the AKT inhibitor LY294002.

**Conclusions:**

This study revealed that the antitumor effects of ARHGAP10 in vivo and in vitro possibly suppress oncogenic glycolysis through the PI3K/AKT/HK2‐regulated glycolysis metabolism pathway.

## 1 INTRODUCTION

Ovarian cancer (OC), a common gynecological malignancy in women, results in over 18 000 deaths each year worldwide and has a poor 5‐year survival rate.^1^, ^2^ Patients are usually diagnosed in late stages, and recurrence often occurs after chemotherapy, targeted therapy, and surgery. However, OC molecular mechanisms are not fully understood. Therefore, it is important to recognize abnormalities in the molecular pathway activity of OC.

Glycolysis is an important feature of solid tumors like OC. More and more research has proven that oncogenes and tumor suppressors regulate energy metabolism.^3^, ^4^, ^5^ Increased glucose uptake and fermentation are the major biochemical events of glucose metabolism in cancer cells. Fermentation converts glucose into pyruvate, eventually producing lactate, which has been proven critical for cancer cell growth.^6^, ^7^, ^8^ Targeting glycolysis has been considered a potential therapeutic strategy in some cancers.^9^, ^10^ Accumulated evidence indicates a relationship between glycolysis and OC cell proliferation and aggressiveness.^11^, ^12^, ^13^ Hexokinase‐2 (HK2), a rate‐limiting enzyme in glycolysis metabolism, is highly expressed in OC.^14^, ^15^ However, little is known about the relationship between ARHGAP10 and HK2 in OC.

Rho GTPase activating protein 10 (ARHGAP10) is located at chromosome 4q31.23.^16^ It consists of the Rho GTPase, which has three domains, the GAP domain, pleckstrin homology (PH) domain, and the PDZ domain.^17^ Circular RNA Rho GTPase‐activating protein 10 (circ‐ARHGAP10) is formed by the exon of the ARHGAP10 gene through circularization and promotes proliferation, migration, invasion, and glycolysis of non‐small cell lung cancer cells.^18^ ARHGAP10 overexpression can inhibit cell proliferation and metastasis, and this function may constrain proliferation and metastasis in human CRC by suppressing the activity of the RhoA/AKT signaling pathway.^19^ Moreover, previous research showed that ARHGAP10 overexpression can inhibit OC cell migration and invasion in vitro.^20^ However, the role of ARHGAP10 in glycolysis and the possible molecular mechanism by which it regulates OC cell migration and invasion needs to be elucidated.

Smad4 belongs to a family of signal transduction proteins. It comprises 552 amino acids and has been implicated as a tumor suppressor.^21^ Previous study has reported that loss‐of‐function alterations in SMAD4 result in TGF‐β being a tumor‐promoting pathway through its interaction with other proteins.^22^ Accumulating evidence indicates a relationship between Smad4 and OC proliferation, invasion, migration, apoptosis, autophagy, or chemoresistance.^23^, ^24^

This study aimed to use two human OC cell lines (OVCAR3 and A2780) and an animal model to explore the role of ARHGAP10 in glycolysis and investigate whether ARHGAP10 affects the expression of HK2 and promotes OC progression.

## 2 MATERIALS AND METHODS

### 2.1 Cell culture, transfection, and lentiviral infection

Two human OC cell lines (OVCAR3 and A2780) were obtained from the Chinese Type Culture Collection, Chinese Academy of Sciences, and maintained at 37°C in a humidified atmosphere containing 5% CO_2_. OVCAR3 and A2780 cells were cultured in RPMI 1640 (Life Technologies, Grand Island, NY) supplemented with 10% fetal bovine serum, 100 U/mL penicillin sodium, and 100 mg/mL streptomycin sulfate.

The sequence of shRNA targeting ARHGAP10 is shown as shARHGAP10, GGTTCACAATTATCAGAAA. The shRNA was included in pLKO.1 lentiviral vectors (Addgene, USA). ARHGAP10 was created and inserted into a pLVX‐Puro plasmid to overexpress ARHGAP10. Following the manufacturer's instructions, lentiviral constructs of pLKO.1‐scramble shRNA (shNC), pLKO.1‐shARHGAP10, pLVX‐Puro empty vector (Clontech, USA), or pLVX‐Puro‐ARHGAP10 were cotransfected with viral packaging plasmids (psPAX2 and pMD2.G) into 293 T cells using lipofectamine 2000 (Invitrogen). The viral supernatant was harvested after 48 h and filtered through a 0.45 μm filter. A2780 cells were infected with pLVX‐Puro‐ARHGAP10 lentivirus, while OVCAR3 cells were infected with pLKO.1‐shARHGAP10 lentivirus in the presence of 8 μg/mL Polybrene. Stable pools were attained with 0.5 μg/mL puromycin (Sigma, St. Louis, MO) and used for the following tests. SMAD4 was created and inserted into pcDNA3.1 plasmid to overexpress SMAD4. Lipofectamine 2000 (Invitrogen) transfected OVCAR3 cells with pcDNA3.1‐SMAD4 or blank pcDNA3.1 as a negative control.

### 2.2 Ethynyl‐2‐deoxyuridine (EdU) assay

Cell proliferation was determined using the EdU assay. Cells were seeded into 24‐well plates and stained according to the instructions of the BeyoClick™ EdU Cell Proliferation Kit (Beyotime Biotechnology, Shanghai) after the abovementioned treatment. Cells were then incubated with a DAPI solution and observed using fluorescence microscopy.

### 2.3 Wound healing assay

Cell migration abilities were evaluated using wound healing assays. Cells were seeded in a 35‐mm culture dish (8 × 10^5^ cells/dish) after the abovementioned treatment. Wounds were created using a sterile pipette tip when cells were fully confluent. After washing with phosphate‐buffered saline to clean the exfoliated cells, they were cultured in a pure medium without fetal bovine serum. The images of wounds at the same position were taken at 0, 24, and 48 h to calculate the distance.

### 2.4 Transwell assay

Stabilization pool cells were cultured in serum‐free medium in the upper chamber of a Matrigel‐coated Transwell (Corning, NY) to determine cell invasion. The lower chamber contained a standard medium with 10% fetal bovine serum. Cells migrating to the submembrane surface were fixed with formalin, stained with 0.05% crystal violet, counted, and imaged under a microscope after 24 h of incubation.

### 2.5 Extracellular flux (XF) analysis

A Seahorse XF24 Extracellular Flux Analyzer determined cellular oxygen consumption rates (OCR) and extracellular acidification rates (ECAR) as previously described.^25^ Briefly, cells digested to a density of 1 × 10^4^/well were seeded in XF‐24 culture plates (Agilent Technologies, Santa Clara, CA) and placed in an incubator at 37°C with 5% CO_2_ for 24 h. Around 1 h before detection, cells were moved to an incubator without CO_2_, and the culture medium was replaced by XF Base Medium (Agilent Technologies). Subsequently, 1 μM oligomycin (ATP synthase inhibitor) was added into “A” well of Seahorse gaging plate, 1.5 μM carbonyl cyanide p‐trifluoromethoxyphenylhydrazone (FCCP; uncoupler) was supplemented into “B” well and then a mixture of antimycin A (complex III inhibitor; 0.5 μM) and rotenone (complex I inhibitor; 0.5 μM) was instilled into “C” well using the Seahorse XF Cell Mito Stress Test Kit (Agilent Technologies). Cellular OCR was monitored using a Seahorse XF24 Extracellular Flux Analyzer. In addition, the cells were treated sequentially with 1 μM of glucose, 1 μM of oligomycin, and 0.5 μM of 2‐Deoxy‐D‐glucose (2‐DG; the glycolytic inhibitor) at time points for measurement of ECAR.

### 2.6 Lactate measurement

Forty‐eight hours after treatment, cells (5 × 10^5^ cells/well) were grown in the six‐well plate, maintained for one day at 37°C. A lactic acid assay kit (Nanjing Jiancheng Bioengineering Institute, China) assessed lactate release following the manufacturer's instructions.

### 2.7 RNA isolation and quantitative RT‐PCR

Trizol reagent (Invitrogen, Carlsbad, CA) was used to extract Total RNA according to the manufacturer's instructions. Quantitative RT‐PCR was used to assess the mRNA levels of ARHGAP10 and SMAD4 using SYBR Green (Thermo Fisher Scientific, Rockford, IL) on an ABI 7500 instrument (Applied Biosystems, Foster City, CA), with GAPDH as an internal control. The primers were: ARHGAP10 (NM_024605.4), 5′‐TGGAGTTCAGCGACTGCTAC‐3′ and 5′‐CTGGGCCACTGACAGACTTT‐3′; SMAD4 (NM_001407041.1), 5′‐CACAAGTCAGCCTGCCAGTA‐3′ and 5′‐AGGCTGGAATGCAAGCTCAT‐3′; β‐actin (NM_001101.5), 5′‐AGGATTCCTATGTGGGCGAC‐3′ and 5′‐ATAGCACAGCCTGGATAGCAA‐3′. All reactions were conducted with the following cycling parameters: 95°C for 10 min, followed by 40 cycles of 95°C for 15 s, 60°C for 45 s. The fold‐change for target genes normalized by internal control was determined by the formula 2^−△△CT^.

### 2.8 Antibodies and western blotting

ARHGAP10 antibodies were obtained from Proteintech Group Incorporated (Rosemont, IL) and HK2, AKT, p‐AKT, SMAD4, and β‐actin antibodies from Abcam (Cambridge, MA). Horseradish peroxidase‐conjugated goat anti‐mouse secondary antibody or goat anti‐rabbit secondary antibody was purchased from ZSGB‐BIO (Beijing, China). Ice‐cold radioimmunoprecipitation assay buffer (50 mM Tris–HCl [pH 7.5], 150 mM NaCl, 1% Triton X‐100, 0.5% Na‐deoxycholate) containing protease inhibitors were used to lyse cells. The BCA protein assay kit (Thermo Fisher Scientific) measured protein concentration. Equal amounts of cell lysates were separated on SDS‐PAGE gels, transferred to PVDF membranes, and analyzed by western blotting using an enhanced chemiluminescence system (Bio‐Rad, Richmond, CA). Image J (NIH, Bethesda, MD) was used to assess band intensities and normalized to β‐actin.

### 2.9 Luciferase activity assay

OVCAR3 cells were transfected with an SMAD4 overexpression vector. Then, wild‐type pGL3‐basic‐ARHGAP10 promoter (WT) or mutant‐type pGL3‐basic‐ARHGAP10 promoter (MUT) luciferase plasmid was transfected into OVCAR3cells. The pRL‐TK vector was transfected into OVCAR3 cells, an internal control reporter. A dual‐luciferase assay was performed according to the manufacturer's protocol. The luciferase activity was evaluated using a Dual‐Luciferase Reporter Assay system (Promega Biotech Co, Ltd, Beijing, China) at 48 h post transfection and normalized to Renilla luciferase activity.

### 2.10 Chromatin immunoprecipitation (ChIP)

Cells were fixed, sonicated, and probed with anti‐SMAD4 (Abcam; ab236321) or control IgG antibody (Cell Signaling Technology; #2729). SMAD4 binding to the ARHGAP10 promoter was detected by PCR with the following primers: 5′‐GGGAAGGGAAGGGAGGAAGAAG‐3′ (F) and 5’‐ATGTCCCGTAGAGCCGAGTC‐3′ (R).

### 2.11 Animal model

The Animal Care and Use Committee of Shanghai Pudong Hospital approved animal experiments. Athymic Balb/c nude mice (4–6 weeks old) were kept in specific pathogen‐free conditions at 25°C with ~50% humidity a regular 12:12‐h light/dark cycle with food and water available ad libitum. After two weeks of acclimatization, mice were injected via the tail vein with OVCAR3 cells transduced with lentivirus expressing shARHGAP10 and intraperitoneal injection of 250 mg/kg/d 2‐DG for 3 weeks. Mice were then sacrificed with an intraperitoneal injection of sodium pentobarbital (30 mg/kg; Vetoquinol UK, Ltd.) followed by cervical dislocation. The lung tissues were collected, and lung metastatic nodules were analyzed with hematoxylin and eosin (H&E) staining (*n* = 6 per group).

### 2.12 Immunohistochemistry (IHC)

The OC tissue microarray, including 40 OC tissues and paired adjacent normal ovarian tissues, was purchased from Shanghai Outdo Biotech (China). Tissues were embedded, sectioned, and stained with an anti‐ARHGAP10 antibody (Proteintech, 55 139‐1‐AP) and anti‐p‐AKT antibody (Abcam, ab38449) followed by an HRP‐conjugated 2nd antibody. Two independent pathologists evaluated the results. Immunoreactivity was scored using the H‐score system by two investigators based on the percentage of positive cells (0–4: 0, <5%; 1, 5%–25%; 2, 25%–50%; 3, 50%–75%; and 4, >75%) and staining intensity [0–3: 0, negative (blue); 1, weak (light yellow); 2, moderate (brown yellow); and 3, strong (dark brown)], ranging from 0 to 12.

### 2.13 Patients and serum samples

Serum samples were from 40 patients with OC admitted to the Shanghai Pudong Hospital. Serum from 40 healthy volunteers was used as a control. The Ethics Committee of Shanghai Pudong Hospital approved the study, and written informed consent was obtained from all patients.

### 2.14 Statistical analysis

GraphPad Prism version 8.4.2 was used for the statistical analysis. Data are presented as the mean ± standard deviation (SD). Comparisons of the two groups were performed with an unpaired Student's *t*‐test. Multiple groups were evaluated using ANOVA followed by Dunnett's multiple comparisons test. Statistically significant differences had a *P*‐value <.05.

## 3 RESULTS

### 3.1 ARHGAP10 inhibits cell migration, invasion, and glycolysis in A2780 cells

**FIGURE 1:**
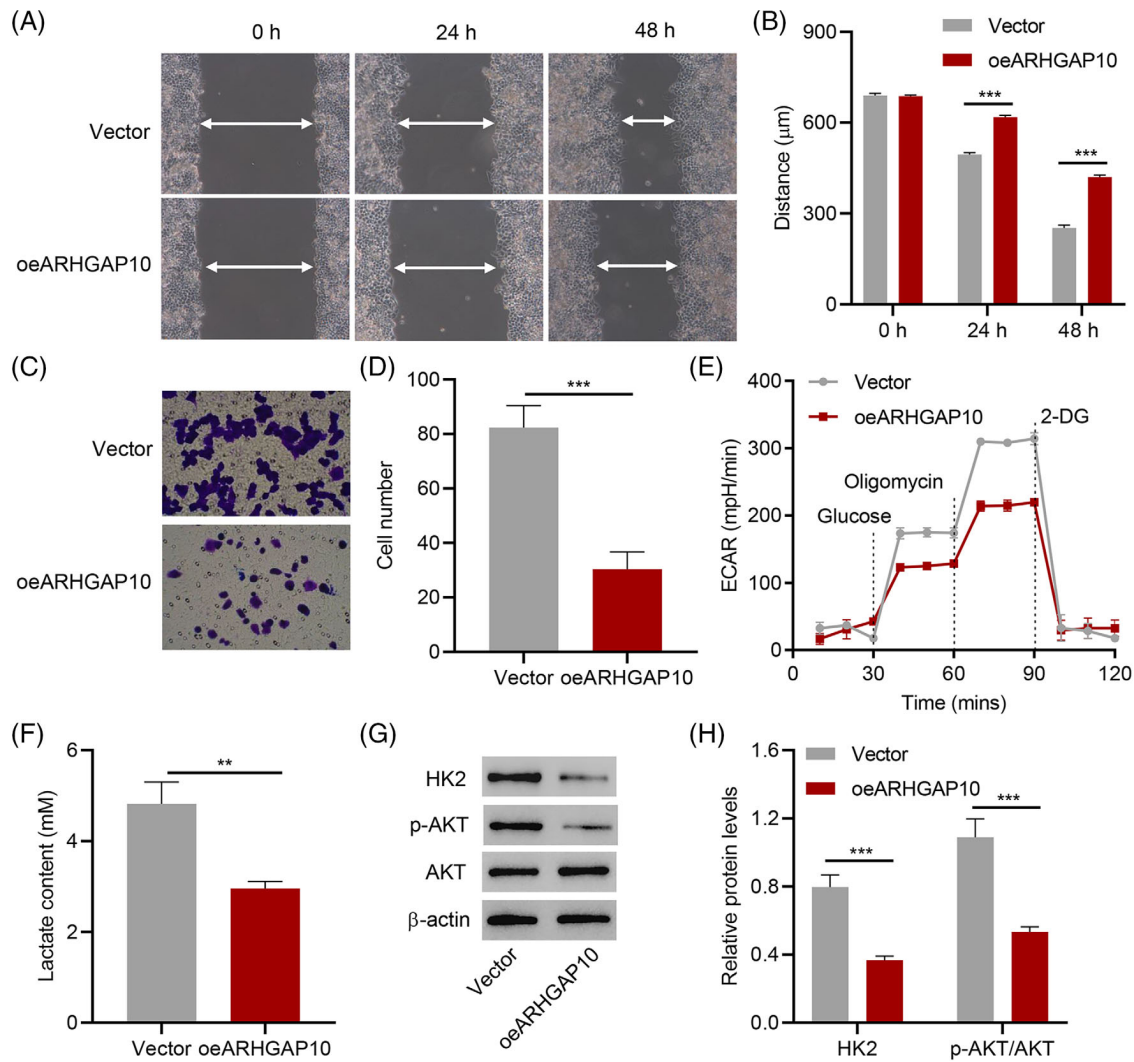
ARHGAP10 overexpression suppressed the proliferation, migration, and glycolysis of A2780 cells. A2780 cells were transduced ARHGAP10 expression vector. (A, B) Cell migration was measured by the wound‐healing assay (arrows in A define the distance in B). (C, D) Cell invasion was measured by the Transwell assay. (E) Glycolysis was measured by ECAR and lactate levels. (G, H) Western blot analysis assessed AKT, p‐AKT, and HK2 expression levels. **P < .01, ***P < .001.

Lentivirus‐mediated ARHGAP10 overexpression was performed in A2780 cells (Supplementary Figure 1A,B) to assess the biological function of ARHGAP10 in OC. ARHGAP10 expression in A2780 cells was lower than other OC cell lines, such as OVCAR3, CAOV3, and SKOV3.^20^ Compared with the control group, ARHGAP10 overexpression significantly suppressed cell proliferation (Supplementary Figure 2A). Meanwhile, ARHGAP10 overexpression remarkably inhibited the migration and invasion of A2780 cells (Figure 1A–D). We tested the ECAR to validate the impact of ARHGAP10 on glucose metabolism. ARHGAP10 overexpression cells had lower ECAR (Figure 1E). Besides, ARHGAP10 overexpression not only decreased lactate production (Figure 1F) but also decreased phospho‐AKT and HK2 levels (Figure 1G). These results indicated that ARHGAP10 can inhibit migration, invasion, and glycolysis of A2780 cells.

### 3.2 Knockdown of ARHGAP10 promotes proliferation, migration, invasion, and glycolysis in OVCAR3 cells via the PI3K/AKT pathway

**FIGURE 2:**
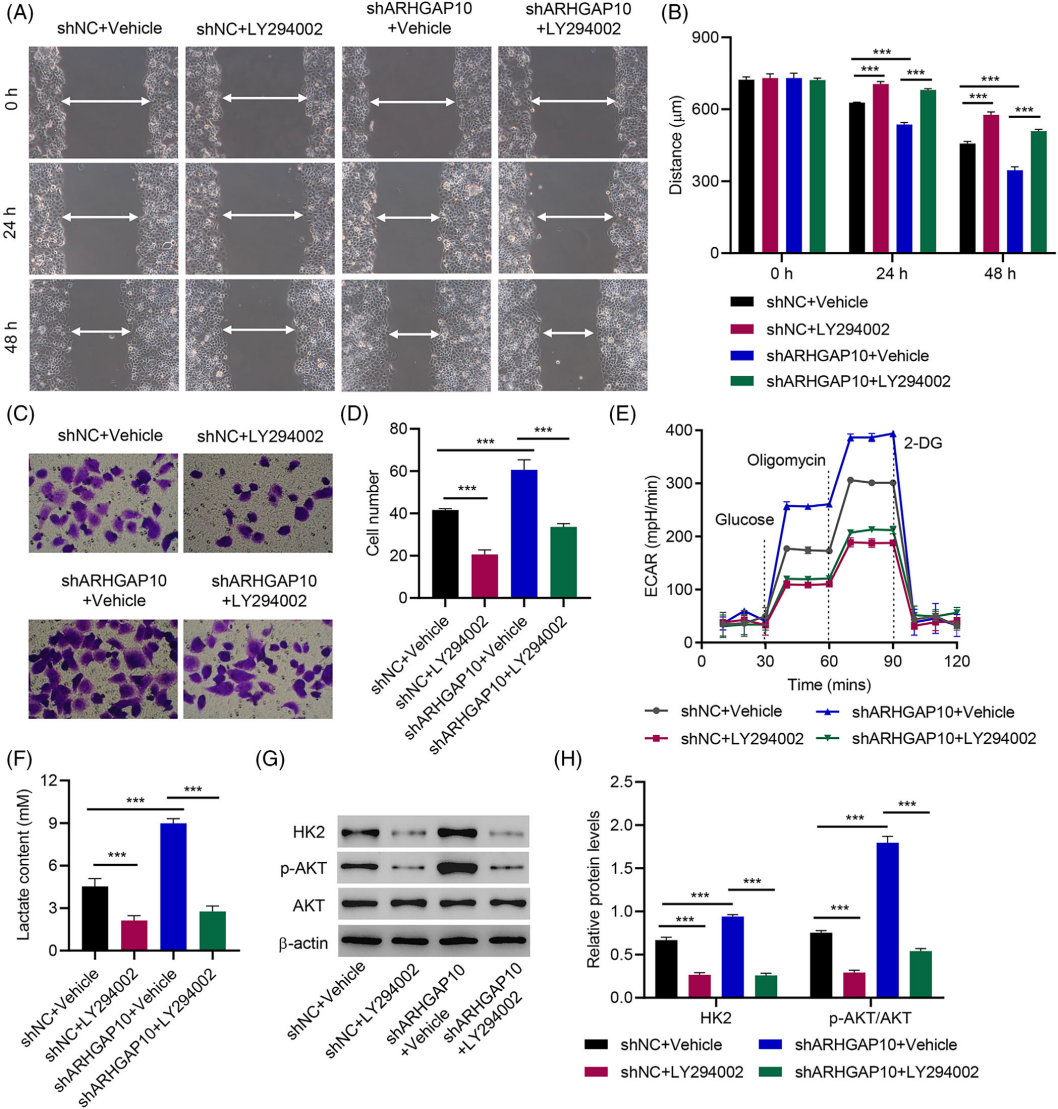
Knockdown of ARHGAP10 promotes proliferation, migration, invasion, and glycolysis in OVCAR3 cells via the PI3K/AKT pathway. OVCAR3 cells were transduced with ARHGAP10 silencing vector and treated with 20 μM LY294002. (A, B) Cell migration was measured by the wound‐healing assay (arrows in A define the distance in B). (C, D) Cell invasion was measured by the Transwell assay. (E) Glycolysis was measured by ECAR and lactate levels. (G, H) Western blot analysis assessed AKT, p‐AKT, and HK2 expression levels. ***P < .001. DMSO acts as the vehicle.

Lentivirus‐mediated silencing of ARHGAP10 was performed in OVCAR3 cells (Supplementary Figure 1C,D) to further assess the biological function of ARHGAP10 in OC. OVCAR3 cells showed higher ARHGAP10 expression than other OC cell lines such as A2780, CAOV3, and SKOV3^20^ with or without 20 μM PI3K/AKT inhibitor LY294002 treatment.^26^ As expected, knockdown of ARHGAP10 significantly promoted OVCAR3 cell proliferation. However, this effect was deeply abolished by the PI3K/AKT inhibitor LY294002 (Supplementary Figure 2B). Furthermore, the knockdown of ARHGAP10 also significantly promoted the migration and invasion of OVCAR3 cells; however, the PI3K/AKT inhibitor LY294002 strongly inhibited the invasion of OVCAR3 cells (Figure 2A–D). Besides, the knockdown of ARHGAP10 increased ECAR with a rise in lactate production (Figure 2E,F). Western blot analysis revealed upregulation of phospho‐AKT and HK2 expression after knockdown of ARHGAP10 in OVCAR3 cells. This phenomenon was inhibited by LY294002 (Figure 2G,H). These data indicate that ARHGAP10 knockdown promotes proliferation, migration, invasion, and glycolysis in OVCAR3 cells via the PI3K/AKT pathway.

### 3.3 Glycolysis inhibition attenuates the effects of ARHGAP10 knockdown on the proliferation, migration, and invasion of OVCAR3 cells

**FIGURE 3:**
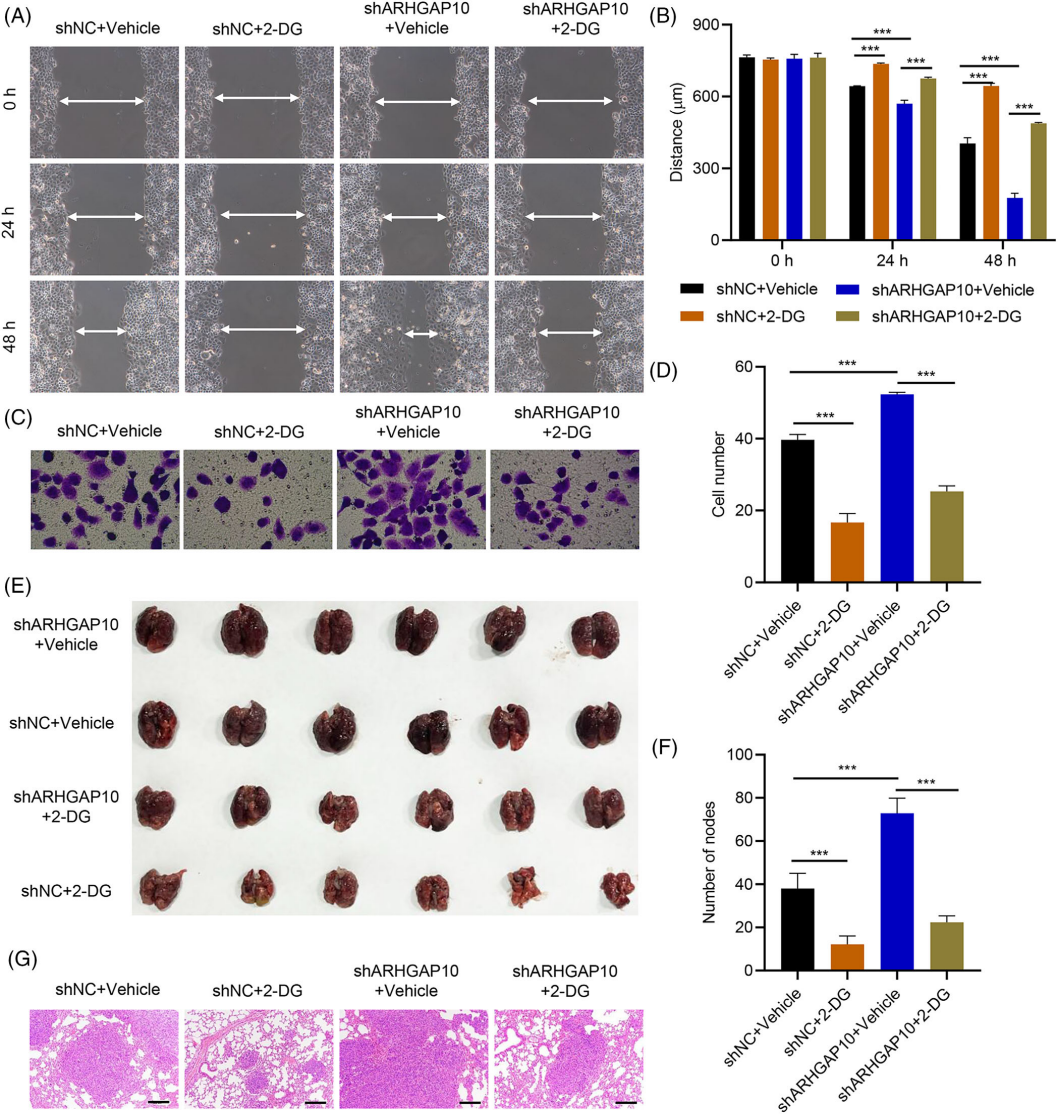
Inhibition of glycolysis attenuates the effects of ARHGAP10 knockdown on the proliferation, migration, and invasion of OVCAR3 cells in vitro and in vivo. (A‐D) OVCAR3 cells were transduced with ARHGAP10 silencing vector and treated with 2.5 mM 2‐DG. (A, B) Cell migration was measured by the wound‐healing assay (arrows in A define the distance in B). (C, D) Cell invasion was measured by the Transwell assay. (E‐G) Mice were injected with OVCAR3 cells with ARHGAP10 silencing and intraperitoneal injection of 250 mg/kg/d 2‐DG for 3 weeks. (E) Representative images of lungs in mice. (F) Quantitative analysis of the lung metastasis nodules in mice. (G) Representative images of H&E staining in mice. Scale bar, 100 μm. ***P < .001. DMSO acts as a vehicle.

We further assessed the mechanism by which ARHGAP10 regulates OC cell proliferation, migration, and invasion. Lentivirus‐mediated silencing of ARHGAP10 was performed in OVCAR3 cells with or without 2.5 mM 2‐DG (a glycolytic inhibitor) treatment.^27^ Furthermore, the EdU, wound healing, and Transwell assay were also examined in OVCAR3 cells after ARHGAP10 knockdown with/without 2.5 mM 2‐DG treatment. ARHGAP10 knockdown combined with 2‐DG inhibited the proliferation (Supplementary Figure 2B), migration (Figure 3A,B), and invasion of OVCAR3 cells induced by ARHGAP10 knockdown (Figure 3C,D). Furthermore, we studied the relationship of ARHGAP10 knockdown with/without treatment with 2‐DG in mice. Our data showed that the mice in the ARHGAP10 knockdown combined with the 2‐DG group had fewer lung metastatic nodules than the ARHGAP10 knockdown group (Figure 3E,F). HE staining of the lungs of these mice showed the same results (Figure 3G).

### 3.4 SMAD4 binds to the core promoter of ARHGAP10 and promotes its expression

**FIGURE 4:**
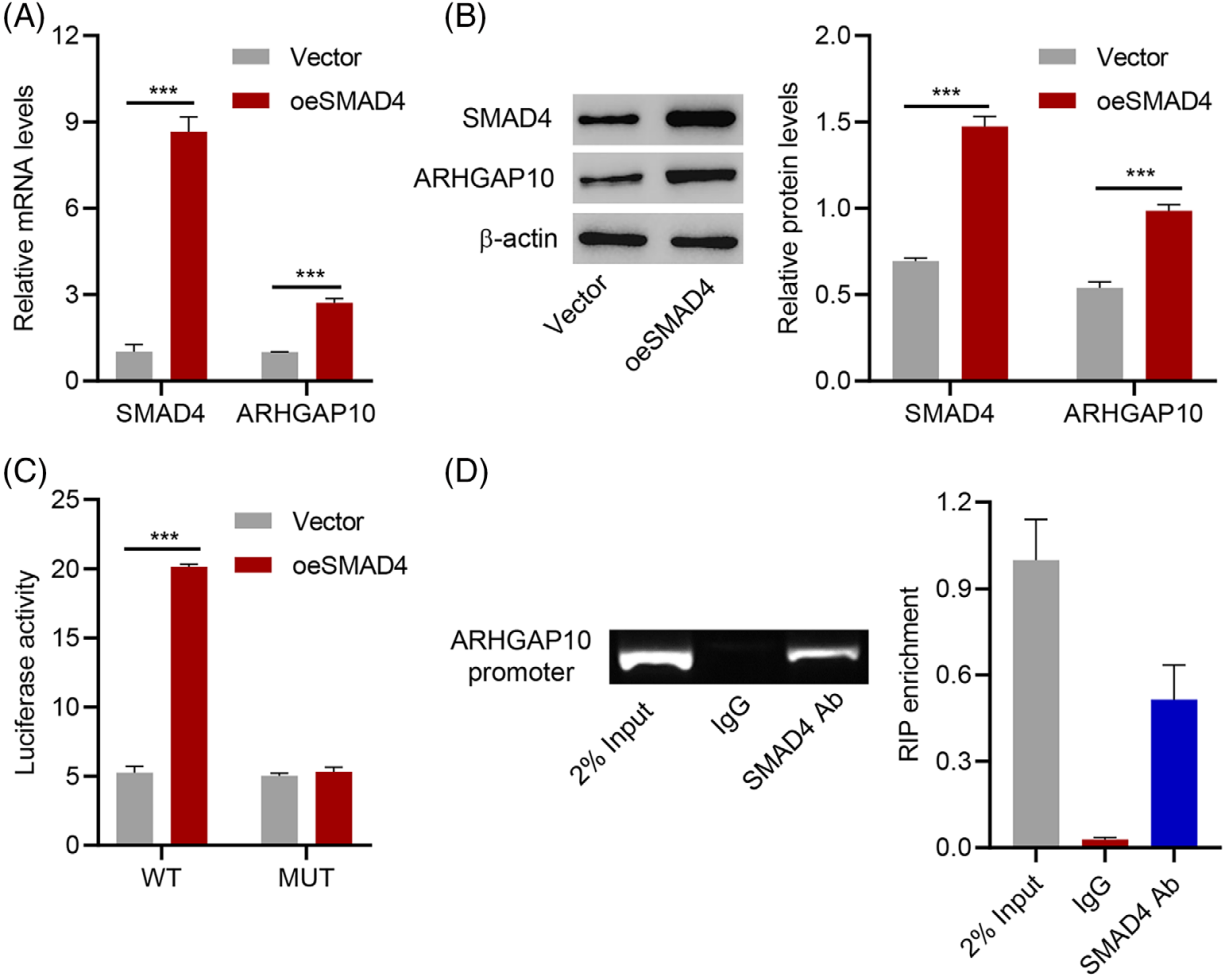
SMAD4 regulates transcription of ARHGAP10. (A) SMAD4 and ARHGAP10 mRNA expression in OVCAR3 cells transfected with the SMAD4 expression vector. (B) SMAD4 and ARHGAP10 protein expression in OVCAR3 cells transfected with SMAD4 expression vector. (C) The Luciferase activity of OVCAR3 cells cotransfected with the wild‐type (WT) or the mutant‐type (MUT) of the ARHGAP10 promoter and SMAD4 expression vector. (D) ChIP results of the binding of SMAD4 to the promoter of ARHGAP10. ***P < .001.

SMAD4, a transcription factor, can regulate gene expression. Therefore, we speculated that SMAD4 induces ARHGAP10 transcription by binding its upstream transcription factor. An SMAD4 overexpression vector was designed and transfected into OVCAR3 cells to determine this fact. Results of qRT‐PCR and western blot analysis illustrated that SMAD4 increased ARHGAP10 expression (Figure 4A,B). Reporter vectors containing 3'UTR wild‐type or 3'UTR mutant‐type ARHGAP10 were constructed and cotransfected with SMAD4 overexpression vector into OVCAR3 cells. SMAD4 overexpression significantly increased the luciferase activity of the ARHGAP10 promoter (Figure 4C). Moreover, chromatin immunoprecipitation (ChIP) assays were performed and revealed that SMAD4 interacts with the promoter of ARHGAP10 (Figure 4D). These results suggest that SMAD4 regulates the transcription of ARHGAP10 by binding to its promoter in OVCAR3 cells.

### 3.5 Correlations between ARHGAP10 and p‐AKT and lactate

**FIGURE 5:**
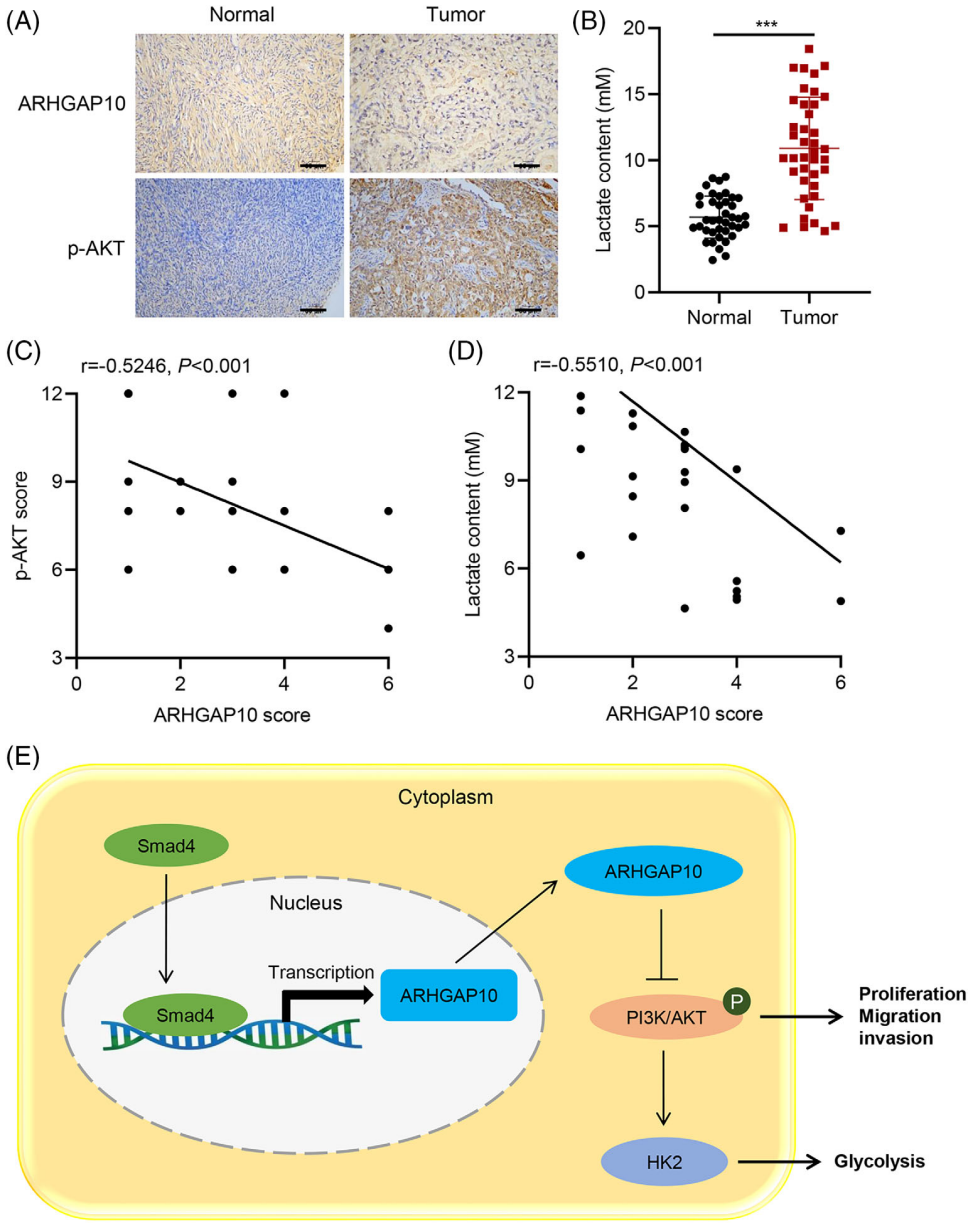
Correlations between ARHGAP10 with p‐AKT and lactate. (A) Representative images of IHC staining in normal and tumor tissues of patients with OC. ARHGAP10 shows weak (light yellow) IHC staining intensity in tumor tissues and moderate (brown‐yellow) IHC staining intensity in normal tissues, respectively; p‐AKT shows strong (dark brown) IHC staining intensity in tumor tissues and weak (light yellow) IHC staining intensity in normal tissues, respectively. Scale bar, 100 μm. (B) The levels of lactate production in the serum of patients with OC and healthy volunteers as normal controls. (C, D) Correlation between ARHGAP10 IHC score and p‐AKT IHC score or lactate content. (E) Schematic representation of the regulation of proliferation, migration, invasion, and glycolysis in OC cells by Smad4‐mediated transcription of ARHGAP10 via the PI3K/AKT/HK2 signaling pathway. ***P < .001.

40 OC tissues and paired adjacent normal ovarian tissues were tested with the IHC assays. ARHGAP10 showed lower IHC score and p‐AKT showed higher IHC score in tumor tissues compared with normal tissues (Figure 5A). Serum lactate production was higher in tumor patients (Figure 5B). Moreover, we further analyzed the correlation between the phospho‐AKT IHC score and lactate production with the ARHGAP10 IHC score. Results suggested that the ARHGAP10 IHC score significantly negatively correlated with the phospho‐AKT IHC score and serum lactate production (Figure 5C,D).

## 4 DISCUSSION

This study demonstrated that ARHGAP10 could constrain the proliferation, migration, and glucose metabolism of OC OVCAR3 cells by inactivating the PI3K/AKT/HK2 pathway. Besides, SMAD4 can regulate this process by upregulating ARHGAP10 expression in vitro (Figure 5E).

ARHGAP10 has been reported to be downregulated in OC tissues and cell lines,^20^ It may also serve as a candidate tumor suppressor in other cancers,^17^, ^28^, ^29^ It played an important antitumor effect in the progression of different cancers through various pathways.^20^, ^30^, ^31^, ^32^ A previous study showed that ARHGAP10 overexpression could suppress cell migration and invasion in OC.^20^ However, the study did not explore the concrete pathway by which ARHGAP10 regulated this process. In non‐small cell lung cancer, emerging evidence has indicated that the antitumor mechanism of ARHGAP10 directly mediated the epithelial‐mesenchymal transition process via the PI3K/Akt/GSK3β pathway.^17^ Although the research was not in OC cells, it provided new insight for us to study the relative antitumor signal pathway of ARHGAP10 in OC. Similar to this study, we found that LY294002 could restore the function of ARHGAP10 in the migration, invasion, and glycolysis of OC cells. Interestingly, ARHGAP10 negatively mediated glycolysis. This discovery was confirmed by the ECAR and lactate production results. Besides, in our research, western blotting results also illustrated that HK2 and p‐AKT were all reduced by ARHGAP10. Most importantly, these results were consistent with the metabolic features of cancer.^12^, ^33^, ^34^, ^35^, ^36^, ^37^ Previous studies have shown that the PI3K/AKT pathway plays an important role in glycolysis.^38^, ^39^, ^40^ Moreover, it has been shown that HK2, one of the key metabolic enzymes of glycolysis, plays a crucial role in OC cell proliferation.^41^, ^42^, ^43^ According to these previous studies, our results demonstrated that the ARHGAP10 is a negative regulator in OC cells, which can downregulate the HK2 and the PI3K/AKT pathways. Therefore, mechanistically, our research elucidated the effect of ARHGAP10 as a tumor suppressor that might inhibit the glycolysis metabolism of OC by inhibiting the PI3K/AKT/HK2 pathway.

Smad4 is involved in various human cancers, and dysregulation of its function is closely related to OC.^44^, ^45^ Recent research has shown that the loss of Smad4 can promote OC metastasis and angiogenesis^46^, ^47^ and predict worse survival in ovarian metastasis patients.^48^ Hence, we further elucidated the relationship between ARHGAP10 and SMAD4. As a direct downstream gene of SMAD4, the result showed that ARHGAP10 expression was significantly upregulated by SMAD4. Previous study has proved that Smad4 can regulate the PI3K/AKT pathway to increase autophagy and apoptosis in ovarian carcinoma cell lines.^44^ Therefore, we speculated that SMAD4 plays a tumor suppressive role by upregulating ARHGAP10 expression and further inhibiting the PI3K/AKT/HK2 pathway.

The innovative aspect of this research was to find the relationship among SMAD4, ARHGAP10, and the PI3K/AKT/HK2 pathway in OC cells, suggesting a new perspective for OC molecular mechanisms research.

There were some pitfalls in our research. First, this was a small clinical sample study lacking relative prognosis information, especially a profound analysis of the correlation between SMAD4 and ovarian patient survival situations. Second, we did not conduct animal models to explore the mechanism of SMAD4 regulating the expression of ARHGAP10, which needs further investigation.

Despite the limited number of clinical samples and in vivo models investigated in our research, our results revealed the underlying molecular mechanism of ARHGAP10 on OC cell proliferation, migration, and glucose metabolism by regulating the PI3K/AKT/HK2 pathway. ARHGAP10 inhibited cell proliferation by suppressing glycolysis mainly through regulation of the key enzyme expression of HK2 and inhibiting the PI3K/AKT pathway. This research highlights the role of SMAD4 in OC progression and provides a new viewpoint for further investigation.

## AUTHOR CONTRIBUTIONS

**Kui Wu:** Writing – original draft (equal). **Wei Gong:** Writing – original draft (equal). **Huanmei Sun:** Data curation (equal); software (equal). **Wenjiao Li:** Data curation (equal); software (equal). **Li Chen:** Methodology (equal). **Yingchun Duan:** Supervision (equal); validation (equal). **jianlong Zhu:** Supervision (equal); validation (equal). **Hu Zhang:** Writing – review and editing (equal). **Huihui Ke:** Conceptualization (lead); resources (lead).

## CONFLICT OF INTEREST STATEMENT

The author(s) declared no potential conflicts of interest with respect to the research, authorship, and/or publication of this article.

## ETHICS STATEMENT

Animal experiments were approved by the Animal Care and Use Committee of Shanghai Pudong Hospital.

## Supporting information


**Supplementary Figure 1.** Evaluation of expression levels of ARHGAP10 in A2780 cells. (A) mRNA and (B) protein expression of ARHGAP10 in A2780 cells transfected with the ARHGAP10 expression vector. (C) mRNA and (D) protein expression of ARHGAP10 in OVCAR3 cells transfected with ARHGAP10 silencing vector. ***P < .001.

**Supplementary Figure 2.** Cell proliferation of OC cells. Cell proliferation of (A) A2780 cells transfected with the ARHGAP10 expression vector and (B) OVCAR3 cells transfected with the ARHGAP10 silencing vector and treated with LY294002 or 2‐DG was measured by the EdU staining assay. Scale bar, 100 μm. **P < .01, ***P < .001.

## Data Availability

All data generated or analyzed during this study are available from the corresponding author upon reasonable request.
