# Supplementary figures and images for: SMAD4 inhibits glycolysis in ovarian cancer through PI3K/AKT/HK2 signaling pathway by activating ARHGAP10
Source: Cancer Rep (Hoboken). 2024 Jan 17;7(2):e1976. doi: 10.1002/cnr2.1976 (PMC10849991; doi:10.1002/cnr2.1976)

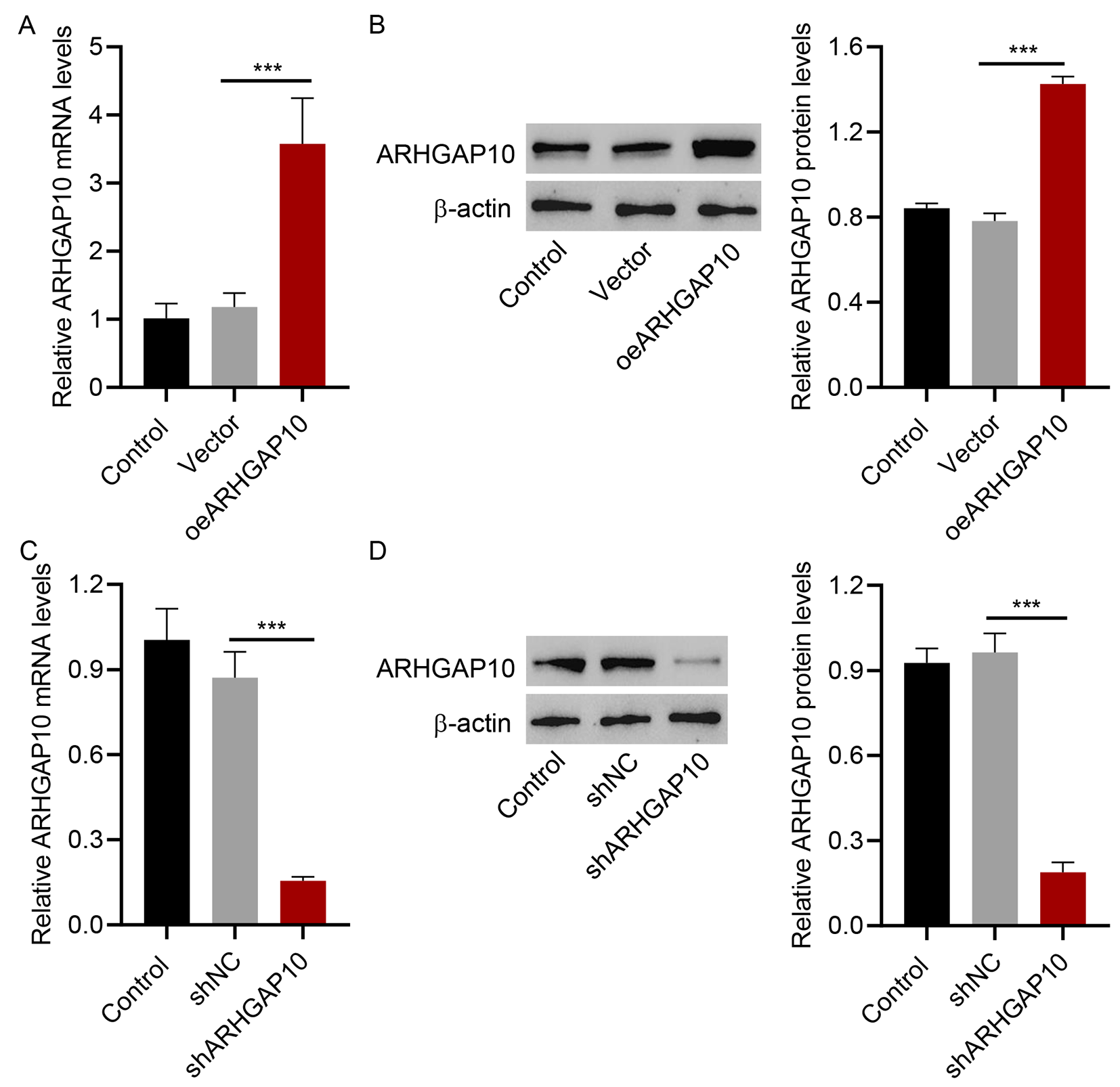

Supplement: Supplementary file 1 — Supplementary Figure 1. Evaluation of expression levels of ARHGAP10 in A2780 cells. (A) mRNA and (B) protein expression of ARHGAP10 in A2780 cells transfected with the ARHGAP10 expression vector. (C) mRNA and (D) protein expression of ARHGAP10 in OVCAR3 cells transfected with ARHGAP10 silencing vector. ***P < .001. [file CNR2-7-e1976-s002.tif]

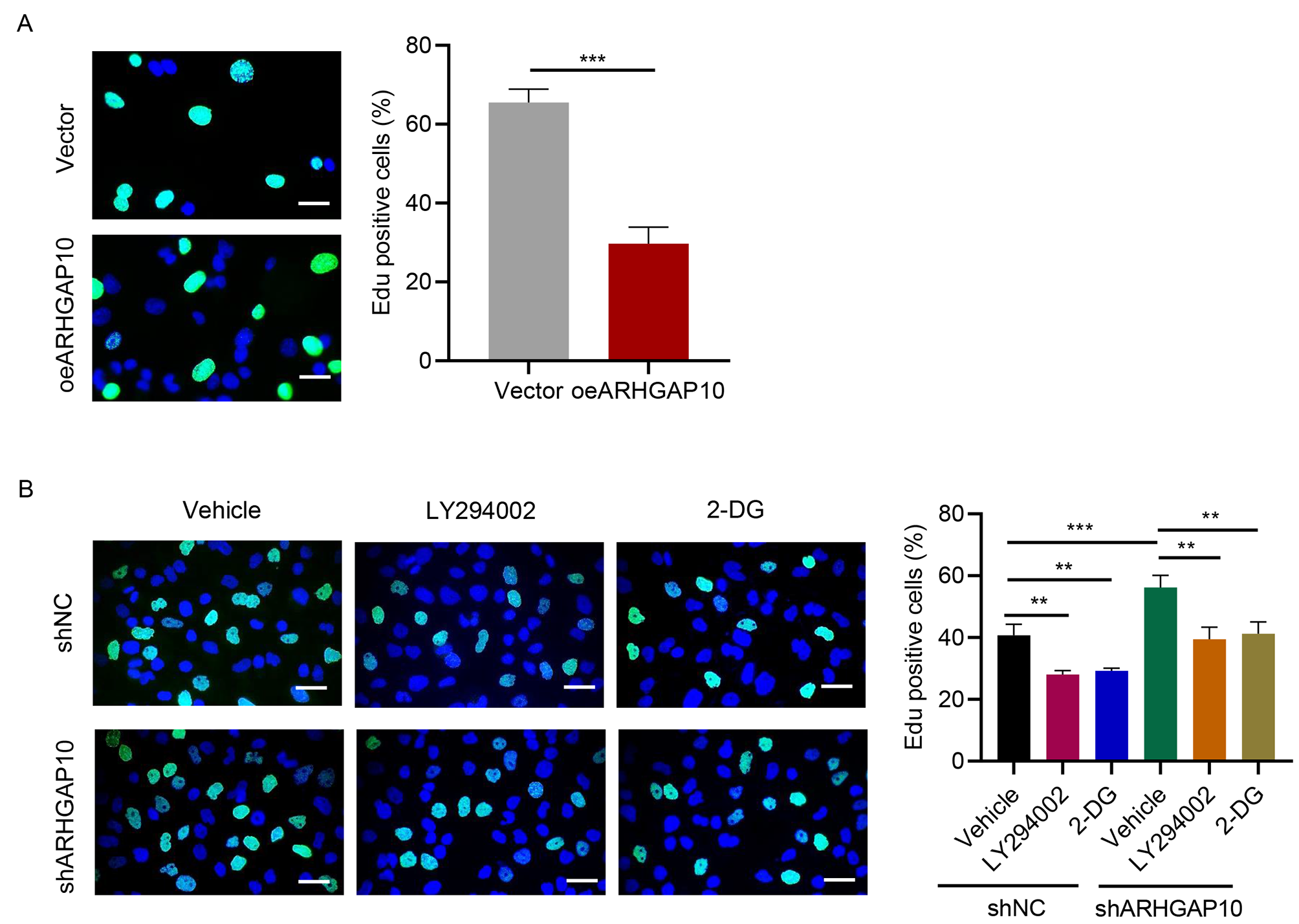

Supplement: Supplementary file 2 — Supplementary Figure 2. Cell proliferation of OC cells. Cell proliferation of (A) A2780 cells transfected with the ARHGAP10 expression vector and (B) OVCAR3 cells transfected with the ARHGAP10 silencing vector and treated with LY294002 or 2‐DG was measured by the EdU staining assay. Scale bar, 100 μm. **P < .01, ***P < .001. [file CNR2-7-e1976-s001.tif]
